# The Role of Fetal Brain Magnetic Resonance Imaging in Current Fetal Medicine

**DOI:** 10.5334/jbsr.3000

**Published:** 2022-12-13

**Authors:** Michael Aertsen

**Affiliations:** 1 KU Leuven, BE

**Keywords:** Fetus, Magnetic Resonance Imaging, Central Nervous system, Cytomegalovirus infection, Diffusion imaging

## Abstract

In open spina bifida we studied the use of MRI for the assessment of the posterior fossa and prevalence of supratentorial anomalies before and after in utero repair. New postprocessing techniques were applied to evaluate fetal brain development in this population compared to controls. In fetuses with congenital diaphragmatic hernia, we evaluated the brain development in comparison to controls. Diffusion weighted imaging was applied to study difference between fetuses with proven first trimester cytomegalovirus infection and controls. Finally, we investigated the value of third trimester fetal brain MRI after treatment for complicated monochorionic diamniotic pregnancies.

## Introduction

Fetal magnetic resonance imaging (MRI) has become an important adjunct to prenatal ultrasound in the assessment of fetal abnormalities of the central nervous system. It has become clear that both modalities are complementary, allowing better understanding of the disease process, classification of abnormalities, and determination of prognosis and management options [1,2,3]. Over the last decades, several in utero treatment options have been proven beneficial and effective, including laser coagulation in monochorionic pregnancies complicated by twin-to-twin transfusion syndrome (TTTS), in utero closure of spina bifida aperta and, most recently fetoscopic endoluminal tracheal occlusion in congenital diaphragmatic hernia (CDH). Furthermore, there have been some recent developments in the prenatal therapy of cytomegalovirus (CMV) infection. The role of imaging of the fetal brain in this population has been recognised widely with an important role for fetal MR [4,5,6].

## Fetal brain MRI in spina bifida aperta

Because of the efficacy of fetal surgery for spina bifida aperta, accurate prenatal imaging of fetuses with spina bifida aperta has become crucial to select eligible fetuses [7]. We investigated the use of fetal MRI in patients being assessed for fetal surgery for spina bifida.

First, we demonstrated that the majority of measurements that are used on postnatal MR images cannot be reliably made around the time of fetal surgery. These measurements include brain stem measurements, and foramen magnum diameter, tentorial length and cisterna magna width. Conversely, assessment of the posterior fossa dimensions and the level of cerebellar herniation were shown reproducible [8]. The latter has been used as a secondary outcome measure in the landmark study on fetal surgery for spina bifida, in other words the Management of Myelomeningocele Study, before and after fetal surgery [9]. Recently the *interpeduncular angle* (IPA) was proven significantly lower in fetuses with open spinal dysraphism, similar to observations in adults with intracranial hypotension [10]. The authors suggest that a reduced IPA is an early sign of CSF-leakage, causing intracranial hypotension, and later followed by frank cerebellar descent.

**Figure 1:**
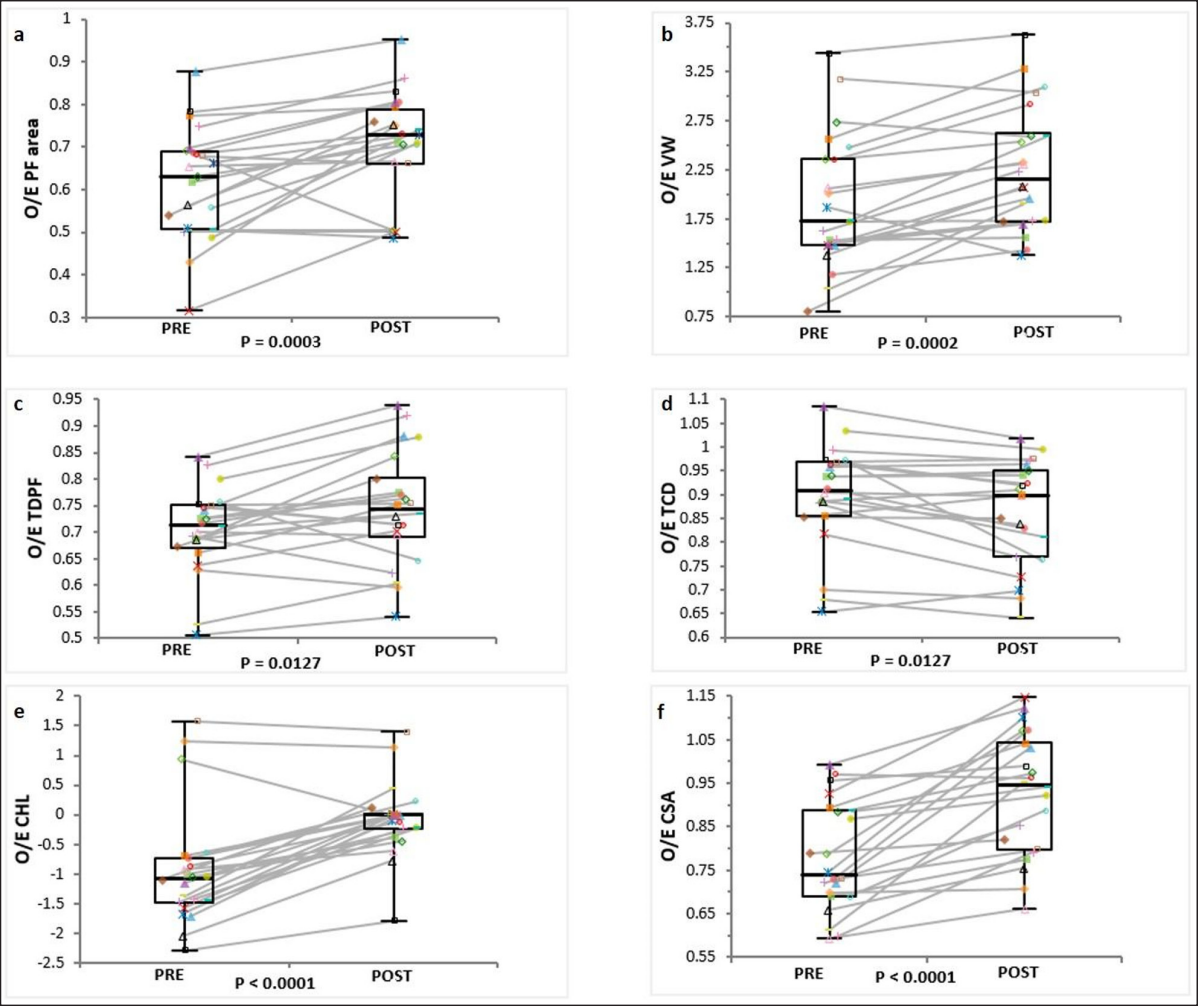
Boxplot demonstrating the minimum, first quartile, median, third quartile and maximum of the observed over expected ratio (O/E) in preoperative open spinal dysraphism (OSD) fetuses and postoperative OSD fetuses at one week for the posterior fossa area (PF area) **(a)**, ventricular width (VW) **(b)**, transverse diameter of the posterior fossa (TDPF) **(c)**, transverse cerebellar diameter (TCD) **(d)**, cerebellar herniation level (CHL) **(e)** and clivus- supraocciput angle (CSA) **(f)**.

In the same study we demonstrated that already within seven days, in the majority of fetuses, there is reappearance of fluid cisterns in the posterior fossa8, Figure 1]. Earlier studies, such as the one from Sutton [11]), also reported such changes between three and six weeks after the surgery in all fetuses where this was measured. The re-accumulation of intracranial CSF can be an interesting proxy of the efficacy of spinal closure [12].

A follow-up MRI later in pregnancy was shown to predict the need for postnatal hydrocephalus treatment [13]. Another reason to perform a postoperative MRI, such as six weeks following surgery, is that the fetal brain can be better documented. For instance, periventricular nodular heterotopia will be more frequently picked up [14,15]. The question is obviously how that information would be used in the further clinical management of the pregnancy, given the advanced gestational age.

Second, we described the nature and occurrence of supratentorial abnormalities in fetuses with spina bifida [16]. Proper assessment is important for counselling women about fetal surgery [17]. Besides ventriculomegaly, evidence of damage to white matter tracts and abnormalities of the corpus callosum [18] or indications of abnormal neuronal migration are frequently observed in fetuses with open spina bifida [14,15,19,20,21,22]. When using MRI in fetuses meeting the criteria for fetal surgery on ultrasound findings, half of them were found to have corpus callosum abnormalities and/or ventricular wall abnormalities [16]. This number is in line with findings in a recent systematic review by our group [23]. Whether MRI is essential for this, hence adds information to US, has to our knowledge not been proven. In our own hands, US also detected a whole range of supratentorial abnormalities. At our center, US findings inform the MRI, and thus we cannot truly measure what would be the theoretical added value of one imaging modality above the other.

Third, we used a new 3D SVR algorithm [24] and an automated segmentation method [25] to document perioperative changes in fetal brain development in fetuses with spina bifida as compared to fetuses without the conditions [26]. Documenting in utero changes following surgery is important, as increasingly fetal surgery is being practiced, and it is expected that more of these operations will be done when minimally invasive methods will be widely implemented [27,28,29]. The shift towards prenatal repair is, at this moment changing the ‘natural history’ of open spina bifida 8,16,30,31,32,33].

In our cohort we did not find any difference in cerebellar volume with that of controls but demonstrated that the cerebellar shape changed importantly after fetal surgery, eventually becoming more comparable to that of controls [26]. The fact that we found cerebellar volumes to be *comparable to that of controls*, needs further investigation. In a prior study, we have found that posterior fossa dimensions in spina bifida prior to 26 weeks were very variable [8]. Others have demonstrated that infants who were not operated in utero, but postnatally, have different white matter and cerebellar volumes. The authors tied this to the mechanical compression and ventricular dilatation present prior to (postnatal) surgery [34,35,36]. The widely accepted theory of McLone and Knepper explains this as follows. The ongoing leakage of CSF at the spinal defect prevents ventricular distention and normal development of the bony structures of the posterior fossa [37]. In turn, the limited growth of the bony posterior fossa limits the cerebellar development, leading to cerebellar compression [35,36].

We also evaluated the white matter in our fetal surgery population, and, again, no differences in volume or shape were found compared to normal controls [26]. These fetuses however had a variable degree of ventriculomegaly prior to fetal surgery; after the operation, the ventricular width continued to increase, in concordance with the observations of others [8,12,16,26]. To us, it remains unclear how the white matter volume evolves during the remainder of the pregnancy and in postnatal life in this subset of patients.

In the same study, we used spectral matching to document cortical folding. We demonstrated an increased shape index prior to fetal surgery, and a decreased shape index seven days after fetal surgery, both compared to the index in normal controls [26]. This is, to our knowledge, the first study specifically documenting cortical development in fetuses before and after fetal surgery. Postnatal studies demonstrated that children with open spina bifida (who underwent postnatal repair) have a different cortical folding pattern in comparison to normal age-matched controls [38,39]. Longitudinal analysis of the cerebellar and white matter development, both volume and shape, as well as cortical folding with spectral matching, will allow us to document the impact of prenatal surgery during the remainder of the pregnancy and potentially also postnatally.

**Figure 2:**
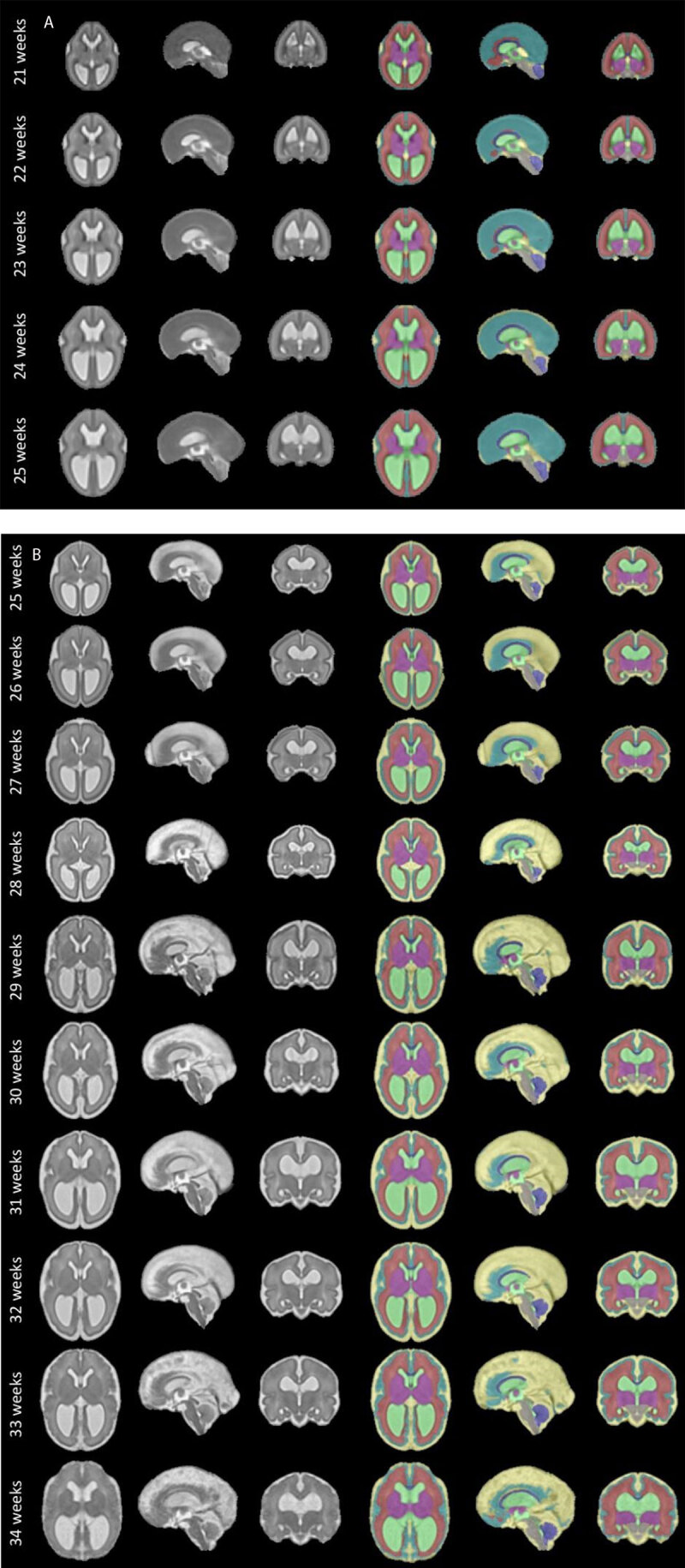
Overview of our Spatio-temporal atlas in fetuses with open spina bifida before **(a)** and after **(b)** in utero spina bifida repair. These include the overview of the eight fetal brain segmentation regions. Yellow: extra-axial cerebrospinal fluid, turquoise: cortex, red: white matter, purple: deep grey matter, grey: brainstem, green: intra-axial cerebrospinal fluid, light blue: cerebellum, dark blue: corpus callosum.

Fourth, we applied the new 3D SVR algorithm [24] to create the first spatio-temporal atlas of the fetal brain in spina bifida aperta [40, Figures 2a, 2b]. The application of our atlas for automated segmentation of fetal brain MRIs with open spinal dysraphism did result in a more accurate segmentation compared to those based on other atlases that used normal fetal brains. This illustrates, as suggested by Jakab et al. [41]. the potential of 3D SVR techniques with automated segmentation. They may eventually provide new outcome predictors for fetuses with this condition based on quantitative research.

## Fetal brain MRI in congenital diaphragmatic hernia

CDH is another congenital malformation for which fetal surgery has been shown to be beneficial in given circumstances [42,43,44]. Imaging studies in infants with CDH have demonstrated several abnormalities, including increased extra-axial space, delayed sulcation and white matter injury, but the exact mechanisms remain unclear, and studies reporting on the neurodevelopmental outcome in isolated CDH are limited [45,46,47]. Fetuses with CDH have variable degrees of hemodynamic dysfunction. We have previously reported a decline of more than 20% in the middle cerebral artery peak systolic velocity, hence in brain perfusion in fetuses with CDH [48]. There may be similarities to the circulatory disturbances in fetuses with congenital heart defects where there are recognized alterations in antenatal brain development. In left-sided CDH, herniation of abdominal structures may result in mild to moderate cardiac hypoplasia, which in turn may compromise cardiac output [49]. Cardiac compression in CDH fetuses may also compromise venous return [49,50]. This may, in turn, cause venous congestion and lead to decreased CSF resorption [51], and an overall increase of intracranial fluid.

We reported on a significant delay in brain development in fetuses with isolated CDH at 28 weeks of gestation and to a lesser extent at 33 weeks of gestation. This is in line with earlier observations by ultrasound and the first MRI data demonstrating an altered brain development in utero in CDH fetuses. Others have not found such differences [50], and those looking only at postnatal data hypothesized that postnatal events may eventually cause altered brain development [50,52,53,54,55,56].

## Fetal brain MRI in congenital cytomegalovirus infection

In fetuses infected with CMV in the first trimester, there is an increased risk of sensorineural hearing loss and impaired cognitive development [1,2,57,58,59,60,61]. Neurosonography (NSG) is the most important modality in the follow-up of fetuses with confirmed first-trimester CMV-infection [59]. We found an added value of fetal MRI in the third trimester in fetuses with proven first-trimester CMV infection [3]. Moreover, there is a correlation between grading of abnormalities found at NSG [62] and those found at MRI [5]. The importance of white matter abnormalities for the outcome is reflected in a new brain MRI score for postnatal evaluation [63]. The only prenatal grading system for brain abnormalities on fetal MRI [5] also includes abnormal white matter hyperintensities. Yet, white matter hyperintensity remains a controversial finding on fetal MRI, especially in cCMV [64,65]. It is probably the most known false-positive finding in CMV because of its subjectivity [5,66]. Recently, Roee et al. [67] have demonstrated MRI detects more subtle findings in CMV-infected fetuses. But more importantly, they found 66% false positive findings in fetuses with an unknown infective status undergoing imaging, the majority being detected on MRI only, emphasizing the importance of amniocentesis in this population [67]. In our study, we evaluated the routine application of diffusion weighted imaging (DWI) in fetuses with proven first-trimester cCMV to evaluate the white matter. Despite a failure rate of >10%, DWI should be implemented in routine fetal MRI for CMV as we found a significant higher ADC value in the brain of cCMV-infected fetuses compared to controls, and our findings suggested a correlation with the severity of abnormalities found on anatomical sequences [3] The higher ADC is in line with a postnatal study comparing cCMV with periventricular leukomalacia (PVL) in children [68].

## Fetal brain MRI in fetuses treated for twin-to-twin transfusion syndrome

**Figure 3:**
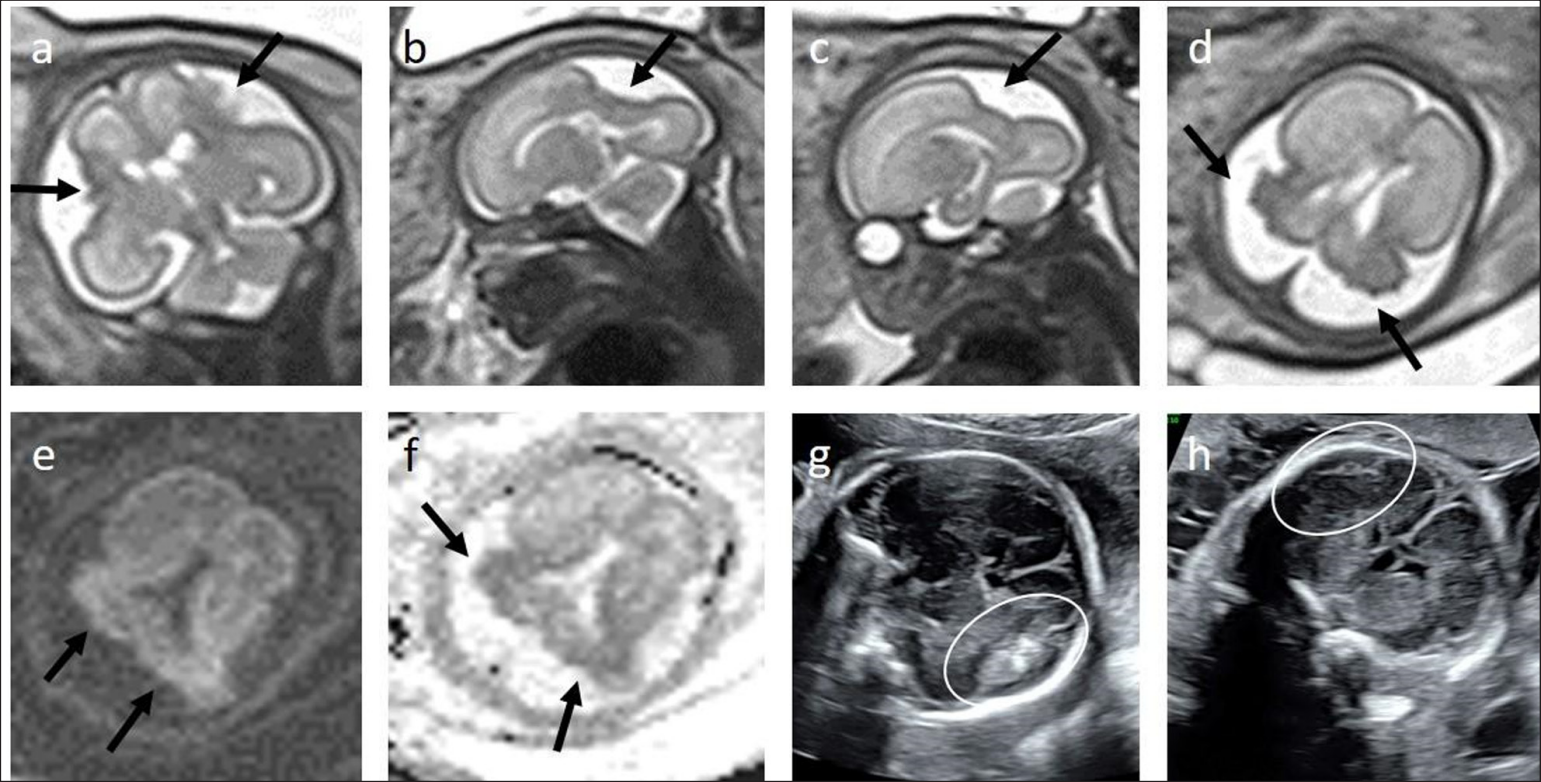
T2-weighted MRI images **(a–d)** and DWI **(e)** with corresponding ADC map **(f)** of an ex-donor at a gestation of 28 weeks (w) in a monochorionic diamniotic pregnancy complicated by TTTS with laser intervention at 20 w (Case 5 in Table 1). Prior US at 24 w **(g)** and 26 w **(h)** are also shown. Fetal MRI was performed for further evaluation of suspected subdural bleeding on the right, which appeared to remain stable in extension but with decreasing echogenicity (white circle in g and h). In addition, the fetus was also monitored closely with US for necrotizing enterocolitis with bowel distension and complicated ascites with peritoneal hyperechoic nodules (not shown). MRI at 28 w (a–f) demonstrates symmetrical bilateral cortical atrophy with irregular lining and hypo-intense signal on T2 (black arrow in a-d), in keeping with old ischemia. On b-1000 of DWI (e) and ADC (f) no acute ischemia was seen; only the same atrophy (black arrow in e and f) was evident.

In twin pregnancies, there is an increased risk of abnormal postnatal neurological development in fetuses surviving TTTS [69,70,71]. Others showed a benefit of fetal brain MRI for the detection of brain abnormalities in TTTS [72,73]. The ISUOG practice guidelines on the role of ultrasound in twin pregnancy do not encourage fetal brain MRI at 30 weeks in survivors after laser ablation TTTS [74]. Nonetheless, we offer our patients a routine fetal brain MRI in the third trimester. In our retrospective study, compared to ultrasound, we found that MRI detected an additional brain lesion in 6% (4/69) [75]. Although the number of abnormalities in our study, as in other studies, was rather small, their consequences however were very important. Of the four pregnancies, the only one that was continued showed cerebral palsy of the affected twin postnatally. The abnormalities only detected on MRI were disorders of cortical development (Table 1), known to be often missed on US and detected more easily on MRI (Table 1) [17,73,76]. Migrational disorders are difficult to diagnose [20,76]. Furthermore, they are more difficult to diagnose on neurosonography, increasing the value of the MRI in these cases [77], (Figure 3). Righini et al. [76] presented their experience in the detection of abnormalities of cortical malformation prior to 24 weeks and described different cortical patterns [76]. Glenn et al. [20] showed that the accuracy of MRI is highest when the abnormality is seen in at least two planes [17]. In addition, we often perform a repeat MRI after 10–14 days for confirmation in these cases. This practice of course is not always feasible if the legislation regarding continuation of the pregnancy is more limited compared to our country [76]. Our results suggest routine third trimester MRI in survivors of TTTS after laser ablation seems justified.

**Table 1:** Characteristics of the six fetuses with brain lesions on third-trimester MRI after laser coagulation of the anastomoses.


	INTERVENTION (WEEKS)	SINGLE IUFD CO-TWIN	DIAGNOSIS	GA MRI (WEEKS)	MAIN LESION	OUTCOME AFFECTED TWIN

Case 1	26.3	No	MRI	29.5; 31.5	Parietotemporal white matter heterogeneity with foci of bleeding; evolution to atrophy on follow-up	Spontaneous in utero demise of donor at 34.5 weeks

Case 2	20.2	No	MRI	30.1	Focal polymicrogyria frontal right	Birth at 34 weeks, cerebral palsy at 5 years

Case 3	16.4	No	MRI	28.2; 31.1	Bilateral focal polymicrogyria	Cord occlusion

Case 4	24.1	Cord occlusion for recurrent TTTS	MRI	27.4	Focal polymicrogyria	Termination of pregnancy

Case 5	19.5	Spontaneous demise day 5 post laser	US + MRI	29.5	Bilateral cortical atrophy secondary to bleeding	Comfort care after birth at 30 weeks because of brain anomaly and prenatal bowel perforation– neonatal demise

Case 6	21.2	No	US + MRI	29.2	Cortical atrophy	Cord occlusion


Legend: GA = gestational age; MRI = magnetic resonance imaging, US = ultrasound; TTTS = twin-to-twin transfusion syndrome; IUFD: Intra-uterine fetal death.

## Conclusion

Although we have demonstrated the added value of fetal brain MRI in several fetal conditions, it remains a challenging technique that needs to be performed upon proven indications and in centers with the necessary expertise in fetal imaging [78]. Not only will this allow maximized exposure in these specialized centers, this will also permit to interpret the findings on fetal imaging (neurosonography and fetal MRI) in a multidisciplinary setting (including fetal specialist, radiologist, pediatric neurologist, geneticist, pathologist) as recommended [78,79,80,81].
